# Postnatal Increases in Axonal Conduction Velocity of an Identified *Drosophila* Interneuron Require Fast Sodium, L-Type Calcium and Shaker Potassium Channels

**DOI:** 10.1523/ENEURO.0181-19.2019

**Published:** 2019-08-05

**Authors:** Dimitrios Kadas, Carsten Duch, Christos Consoulas

**Affiliations:** 1 Laboratory of Experimental Physiology, National and Kapodistrian University of Athens, Athens 11527, Greece; 2 Institute of Developmental Biology and Neurobiology, Johannes Gutenberg University Mainz, Mainz 55122, Germany

**Keywords:** action potential propagation, escape, giant fiber, insect, postnatal maturation, voltage-gated ion channels

## Abstract

During early postnatal life, speed up of signal propagation through many central and peripheral neurons has been associated with an increase in axon diameter or/and myelination. Especially in unmyelinated axons postnatal adjustments of axonal membrane conductances is potentially a third mechanism but solid evidence is lacking. Here, we show that axonal action potential (AP) conduction velocity in the *Drosophila* giant fiber (GF) interneuron, which is required for fast long-distance signal conduction through the escape circuit, is increased by 80% during the first day of adult life. Genetic manipulations indicate that this postnatal increase in AP conduction velocity in the unmyelinated GF axon is likely owed to adjustments of ion channel expression or properties rather than axon diameter increases. Specifically, targeted RNAi knock-down of either Para fast voltage-gated sodium, Shaker potassium (*Kv1* homologue), or surprisingly, L-type like calcium channels counteracts postnatal increases in GF axonal conduction velocity. By contrast, the calcium-dependent potassium channel Slowpoke (BK) is not essential for postnatal speeding, although it also significantly increases conduction velocity. Therefore, we identified multiple ion channels that function to support fast axonal AP conduction velocity, but only a subset of these are regulated during early postnatal life to maximize conduction velocity. Despite its large diameter (∼7 µm) and postnatal regulation of multiple ionic conductances, mature GF axonal conduction velocity is still 20–60 times slower than that of vertebrate Aβ sensory axons and α motoneurons, thus unraveling the limits of long-range information transfer speed through invertebrate circuits.

## Significance Statement

An effective mechanism to increase information processing speed through neural circuits is enhancing plasma membrane insulation through optimizing myelination, a means that cannot be capitalized on un-myelinated invertebrate axons. We identify postnatal adjustments of the expression levels of fast sodium, Shaker potassium, and L-type calcium channels as a mechanism to almost double axonal conduction velocity in the *Drosophila* giant fiber (GF) axon, the core component for long-range information transfer through a neural circuit optimized for fast escape responses. However, despite the regulation of multiple ion channels, mature GF axonal conduction velocity still lacks magnitudes of order behind that of myelinated vertebrate fibers, thus hinting to long distance information transfer as a size-constrain in the evolution of invertebrate circuits.

## Introduction

CNS development is not completed at birth, but early postnatal life is accompanied by structural and physiologic refinement of neuronal circuits to adjust function to the new life conditions (Webb et al., 2001). In mammals, the first two to three weeks after birth are characterized by changes in synapse number (Kano et al., 2018) and strength (Maffei and Turrigiano, 2008) as well as changes in passive and active membrane properties to adjust network activity (Fulton, 1986; Viana et al., 1994; Zhou and Hablitz, 1996; Vincent and Tell, 1997; Cabanes et al., 2002).

Moreover, in many central and sensory neurons axonal action potential (AP) conduction velocity is increased postnatally, thus accelerating the speed of long rang information transfer through the nervous system (NS). Mis-regulation of this mechanism has been linked to neurologic and psychiatric disorders, such as epilepsy and schizophrenia (Scharfman and McCloskey, 2009; Jaaro-Peled et al., 2009; Duncan et al., 2010; Pun et al., 2012). In the vertebrate NS, axonal conduction velocity is most effectively increased by myelination (Peters and Muir, 1959; Gibson et al., 2014), and this process extends far into postnatal life (Barnea-Goraly et al., 2005). In unmyelinated axons, conduction velocity can potentially be regulated by changes in diameter, but up-regulation is limited by space constraints. As an additional mechanism, changes in axonal ionic conductances have been suggested (Foster et al., 1982; Fitzgerald, 1985, 1987; Fulton, 1987). Although the spatial and temporal patterns of ion channel expression are regulated differentially during NS postnatal development, the contributions of these processes to adjustments of axonal conduction velocity are incompletely understood.

We employ a combination of *Drosophila* genetics and electrophysiology to unravel ionic mechanisms that cause postnatal increases in AP propagation speed in an identified interneuron of the giant fiber system (GFS). The GFS is an anatomically and electrophysiologically well characterized neural circuit which mediates the jump-and-flight escape reflex in response to a threatening stimulus. The large diameter GF interneuron receives sensory input in the brain and relays this information via a descending axon to the escape motor circuit in the ventral nerve cord (King and Wyman, 1980; Tanouye and Wyman, 1980; Trimarchi and Schneiderman, 1993; Hammond and O’Shea, 2007). Therefore, axonal conduction velocity through the GF axon is critical for fast escape.

We demonstrate that postnatal adjustments of ion channel expression in the GF interneuron increase axonal conduction velocity by 80% during the first day of adult life. Our data indicate that increases in the expression of fast voltage-gated sodium, Shaker potassium (*Kv1* homolog), and to our surprise, L-type like calcium channels mediate postnatal increases in GF axonal conduction velocity. Moreover, other active conductances, such as the BK channel, Slowpoke, also increase conduction velocity, but they do not substantially affect postnatal speeding. Therefore, we identified multiple ion channels that function to support fast axonal AP conduction velocity, but only a subset of these are regulated during postnatal life to tune the unmyelinated GF axon to maximum speed.

## Materials and Methods

### *Drosophila* strains and culture

Flies were raised on standard corn flour-yeast-agar medium at 24°C in a humidified incubator. Adult 1 h post-eclosion (1hPE) and 24 h post-eclosion (24hPE) flies of both sexes were used for all experiments. The *GF-split-Gal4* strain [*17A04_p65ADZp (attp40); 68A06_ZpGdbd (attP2)*], which drives expression in the two GF interneurons only (von Reyn et al., 2014), was crossed to the following strains containing UAS insertions: *y^1^ v^1^; P{UAS-GFP.VALIUM10}attP2* used as a control (Bloomington Stock Center, 35786; RRID:BDSC_35786), y^1^ sc* v^1^; P{TRiP.HMS00868}attP2 that expresses dsRNA for RNAi of *para* (Bloomington Stock Center, 33923; RRID:BDSC_33923), y^1^ sc^*^ v^1^; P{TRiP.HMS00294}attP2 that expresses dsRNA for RNAi of *DmCa1D* (Bloomington Stock Center, 33413; RRID:BDSC_33413), y^1^ sc^*^ v^1^; P{TRiP.HMC03576}attP40 that expresses dsRNA for RNAi of *shaker* (Bloomington Stock Center, 53347; RRID:BDSC_53347), y^1^ sc^*^ v^1^; P{TRiP.HMS05837}attP40 that expresses dsRNA for RNAi of *shal* (Bloomington Stock Center, 67976; RRID:BDSC_67976), y^1^ sc^*^ v^1^; P{TRiP.HMC04093}attP40 that expresses dsRNA for RNAi of *slowpoke* (Bloomington Stock Center, 55405; RRID:BDSC_55405), y^1^ w^*^; PUAS-NaChBac-EGFP}1/TM3, Sb^1^ that expresses EGFP-tagged bacterial sodium channel (NaChBac; Bloomington Stock Center, 9467; RRID:BDSC_9467). For light microscopic analysis of Shaker channels along the GF axon, a protein trap fly strain (Bloomington Stock Center, 59423; RRID:BDSC_59423) with endogenously GFP-tagged Shaker channels (y^1^w^*^ Mi{PT-GFSTF.2}Sh^MI10885-GFSTF.2^) was recombined with the GF-split-Gal4 line and UAS-cd4-tomato (*w;GMR17A04-pBPp65ADZpUw attP40 UAS-cd4::td-tomato;GMR68A06-pBPZpGAL4DBDUw attP2*). The homozygous recombinant expresses GFP tagged shaker channels and UAS-cd4-tomato in the GF.

### Effectiveness of RNAi constructs used

Although we have not measured the RNAi knock-down efficacy in the GF interneuron, four out of the five UAS-RNAi constructs used in this study have previously been validated in *Drosophila* motoneurons. The RNAi for DmCa1D calcium channels (Bloomington Stock Center, 33413; RRID:BDSC_33413) causes a 70% reduction on larval motoneuron L-type calcium current as measured by somatic voltage clamp recordings (Kadas et al., 2017). This has further been confirmed by immunocytochemistry for Dmca1D in larval motoneuron somata (Kadas et al., 2017) and by calcium imaging (Schützler et al., 2019). UAS-slo-RNAi (Bloomington Stock Center, 55405; RRID:BDSC_55405) has been tested in with the same Gal4 driver in the same types of larval motoneurons and causes a reduction in slow mediated transient outward current by ∼50% (Kadas et al., 2015). Therefore, we expect knock-down efficacy of slo-RNAi in the GF to be slightly lower than that of Dmca1D-RNAi. The UAS-Shal RNAi construct has been validated to cause >80% of shal-mediated outward current by electrophysiological studies in the same larval motoneurons (Schaefer et al., 2010), and knock-down efficacy has been further confirmed in adult *Drosophila* motoneurons (Ryglewski and Duch, 2009). For UAS-para-RNAi knock-down efficacy has not been quantified in *Drosophila* neurons. However, effectiveness and specificity have been validated indirectly by showing that UAS-para-RNAi has similar effects like para hypomorphic mutations (Kaas et al., 2016).

### Electrophysiological preparation and recordings

Flies were anesthetized briefly glued to a thin metal wire attached to the neck with cyanoacrylate adhesive allowed to recover from anesthesia at least for 30 min. To stimulate electrically the GF neurons or thoracic motoneurons, a pair of uninsulated tungsten electrodes was used to penetrate the eyes or thorax, respectively. A similar electrode was used to record from the dorsal longitudinal flight muscle (DLM)5–6 or tergotrochanteral muscle (TTM). A fourth tungsten reference electrode was placed into the scutellum or the abdomen (Kadas et al., 2012).

Brain stimulation was performed by delivering stimuli (0.15 ms in duration) with a Grass S88 stimulator, while DLM or TTM muscle APs were acquired in the 300-Hz to 10-KHz frequency range and amplified 100× by a differential AC amplifier (A-M Systems model 1700). Data were digitized with an analog-to-digital converter (Digidata 1200, Molecular Devices) and without filtering were analyzed and displayed with Clampex 8.1 version software (Molecular Devices).

### Confocal microscopy

All images were acquired with a Leica SP8 confocal laser scanning microscope (Leica Microsystems Inc, RRID:SSR_004098) with excitation wavelengths at 488 nm (Argon laser) and at 561 nm (DPSS laser). Detection was conducted with photomultipliers at wavelengths between 495 and 515 nm and between 570 and 600 nm, respectively. GF diameter was measured live in freshly dissected animals with a 63× water dipping lens and a z-step size of 1 µm. Co-localization analysis of GFP-tagged shaker channels and the GF interneuron was conducted in fixed and cleared preparations using a 40× oil lens (NA: 1.25). Maximum magnification used was zoom factor 3.5, and 290-nm z-step distances, thus yielding voxel dimensions of 86 × 86 × 290 nm (*x*, *y*, *z*).

### Statistical analysis

Statistics were performed with GraphPad Prism 6.00 for Windows. Data were tested for normality (D’Agostino and Pearson omnibus normality test) and unpaired *t* test was used to compare between pairs. Data were presented as means ± SEM, and significance levels were defined as *p < 0.05; **p < 0.01, ***p < 0.001, and ****p < 0.0001.

## Results

### GF conduction velocity increases over the first day of adult life

**Figure 1.**
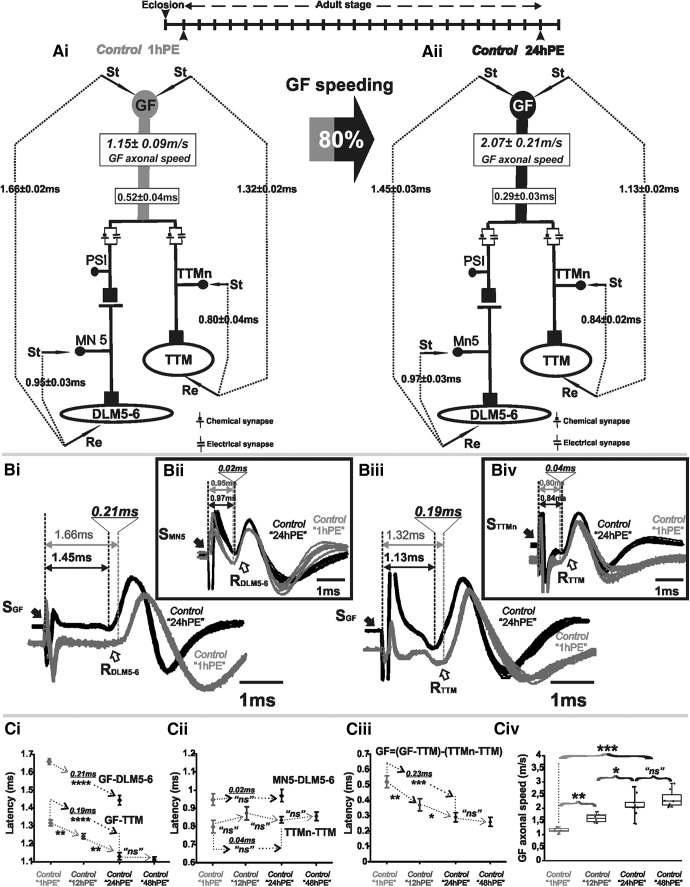
The GF axonal conduction velocity increases 80% during postnatal maturation. ***A***, GFS schematic depiction at 1hPE (***Ai***) and 24hPE (***Aii***) control flies. Average latency ± SEM for GF-DLM5–6, GF-TTM, MN5-DLM5–6, and TTMn-TTM branches are indicated between dotted lines. Lower boxes on GF indicate the latency in the GF axon. Upper boxes on GF indicates the GF axonal speed. The percentage of the GF speeding (80%) during the first day of the fly life is presented in the gray-black arrow. ***B***, Ten overlapping sweeps of APs recorded from DLM5–6 muscles, after GF (***Bi***) or MN5 (***Bii***) stimulation, and from TTM muscle, after GF (***Biii***) or TTMn (***Biv***) stimulation, at 1hPE (gray) and 24hPE (black) flies. Times above double arrows indicate the latency, which is measured as the interval of time between the stimulus artifact (black arrow) and the onset of the initial phase of muscle potential (white arrow). Underlined time values show the difference in latency measurements between the two stages. ***C***, Latency measurements in the GF-DLM5–6 and GF-TTM pathways (***Ci***), in the MN5-DLM5–6 and TTMn-TTM sub-pathways (***Cii***), in the GF axon (***Ciii***), and measurements of the GF axonal speed (***Civ***), at 1hPE (50% black), 12hPE (70% black), and 24hPE or 48hPE (black) flies. Underlined time values between dotted arrows indicate the latency difference between1hPE and 24hPE. Data are shown as means ± SEM (***Ci–Ciii***). Dots on box plots showcase the measurements from individual flies (***Civ***). Asterisks indicate *p* values from one-way ANOVA with *post hoc* Dunnett’s tests (*p < 0.05, **p < 0.01, ***p < 0.001, ****p < 0.0001, n.s., *p* > 0.19). St, stimulation site; Re, recording site; S, stimulus; R, record.

On each side of the *Drosophila* CNS the GFS consists of a GF interneuron that receives sensory information to its dendrites located in the brain and relays this information through a descending axon to two thoracic motor sub-circuits: the GF-TTM system, controlling the extension of mesothoracic legs during escape jumping, and the GF-DLMs system, controlling wing downstroke during flight initiation (Fig. 1*A*). In the GF-TTM and GF-DLM pathways, the GF makes mixed electrical and chemical (cholinergic) synapses with the TTM jumping motoneuron (TTMn) and the peripherally synapsing interneuron (PSI; Phelan et al., 1996; Sun and Wyman, 1996; Allen et al., 1999; Blagburn et al., 1999; Allen and Murphey, 2007). The PSI synapses directly onto the axons of the five motoneurons (MN1–5) that innervate the DLMs. MN1–4 innervate the ventral-most four DLM fibers, whereas MN5 innervates the two dorsalmost DLM fibers (Fig. 1*A*; King and Wyman, 1980; Koto et al., 1981; Ikeda and Koenig, 1988; Sun and Wyman, 1997; Koenig and Ikeda, 2005; Allen et al., 2006). The GF to DLM flight muscle pathway comprises one synapse (PSI to MN1–5) more than the GF to TTM pathway (Fig. 1*A*), thus adding the time for one chemical synaptic transmission onto the signal delay between GF and muscle. Therefore, the typical escape response is a jump followed by flight.

To investigate whether the GF interneuron and/or the downstream motor circuits are subject to functional changes during early postnatal life, circuit performance was compared by electrophysiological recordings in newly eclosed (1hPE) and 1-d-old mature flies (24hPE). We bypassed all sensory neurons and synaptic input computation times in the GF dendrites by direct electrical stimulation of the GF interneuron. Electrical stimulation of the GF elicits a muscle potential in the TTM and in the DLM5–6 muscles (Fig. 1*Ai*,*Aii*). The response latency (the time between GF stimulation and muscle potential; Fig. 1*B*) is the sum of the durations for AP conduction through the GF, PSI, and respective motoneuron axons, plus the time for synaptic transmission. As stated above, for the DLM branch, the PSI to MN5 synaptic delay adds to this latency (Tanouye and Wyman, 1980; Engel and Wu, 1992; Kadas et al., 2012). Therefore the latency of the GF-DLM5–6 (1.45 ± 0.03 ms) pathway is significantly (statistical test and *p* values are provided in figure legends) longer than that of the GF-TTM (1.13 ± 0.02 ms) pathway (Fig. 1*Aii*,*Bi*,*Biii*,*Ci*), although the net effect size is only ∼0.3 ms, indicating that fast chemical synaptic transmission between PSI and MN5 takes only about one third of a millisecond. Please note that synaptic transmission between the GF and the TTMn is dominated by the electrical component of the mixed synapse. If the electrical component is blocked, the chemical component of the GF/TTMn synapse increases the latency in the GF-TTM pathway almost to the level of the GF-DLM5–6 pathway (Allen and Murphey, 2007; Pézier et al., 2016).

Comparing the response latency of the GF-DLM5–6 branch between 1-h-old and 1-d-old flies reveals a significant shortening during the first day of postnatal life from 1.66 ± 0.02 ms at 1hPE to 1.45 ± 0.03 ms at 24hPE (Fig. 1*Ai*,*Aii*,*Bi*,*Ci*). Similarly, 1-d-old flies exhibit a shorter GF-TTM latency response (1.13 ± 0.02 ms) in comparison to 1-h-old flies (1.32 ± 0.02 ms; Fig.1*Ai*,*Aii*,*Biii*,*Ci*). Therefore, during the first day of adult life (1–24hPE), response latency of both branches of the GFS is decreased by ∼0.2 ms, which equals to an improvement of 14% for the GF-DLM branch (0.21 ms) and 17% for the GF-TTM branch (0.19 ms; Fig. 1*Bi*,*Biii*,*Ci*). Additional measurements at 48 h post-eclosion indicated that postnatal maturation was completed by 24 h because response latency did not further decrease at 48 h (Fig. 1*Ciii*,*Civ*). Measurements at 12 h revealed intermediated values as compared to 1 and 24 h post-eclosion (Fig. 1*Ciii*,*Civ*)., thus indicating that conduction velocity is likely steadily increased during the first day of adult life. Although net effect size is only ∼0.2 ms, this increase in conduction velocity may be of biological relevance considering that it is close to the normal difference of information processing speed through the GF-TTM versus the GF-DLM path, which ensures that jumping precedes flight initiation (The GF to DLM flight muscle pathway comprises one synapse (PSI to MN1–5) more than the GF to TTM pathway (Fig. 1*A*), thus adding the time for one chemical synaptic transmission onto the signal delay between GF and muscle. Therefore, the typical escape response is a jump followed by flight.). A similar net decrease in latency in both GFS branches seems important to maintain the time difference in jump and succeeding flight initiation, and it indicates functional maturation of common circuit elements (axons/synapses). We next aimed at pinpointing the cellular site that underlies the postnatal increases in information transfer.

To test for potential maturation of the motoneurons and/or the neuromuscular synapses, we stimulated the motoneurons directly by inserting tungsten electrodes into the thoracic nerve cord and recorded the responses from the DLM and the TTM muscle, respectively. The latency between motoneuron activation and muscles responses of 1-h-old flies was not significantly different from that of 1-d-old flies, for both, the DLM branch (1hPE, 0.95 ± 0.03 ms and 24hPE, 0.97 ± 0.03 ms; Fig. 1*A*,*Bii*,*Cii*) and the TTM branch (1hPE, 0.80 ± 0.04 ms and 24hPE, 0.84 ± 0.02 ms; Fig. 1*A*,*Biv*,*Cii*). Hence, motoneuron axonal conduction speed and neuromuscular transmission delay do not undergo postnatal changes. This leaves GF axonal conduction speed as cause for postnatal speeding of GF.

The AP conduction time in the GF axon can be estimated by subtracting the TTMn-to-TTM latency from the GF-to-TTM latency. Note that the GF to TTMn synapse is dominated by electrical transmission and, thus, does not add notable time to the latency. According to this calculation the GF axonal conduction duration decreased by 80% during the first day of postnatal period, from 0.52 ± 0.04 ms in 1hPE flies to 0.29 ± 0.03 ms in 24hPE flies (∼0.23 ms; Fig. 1*A*,*Ciii*; Table 1). Considering that the axon of the GF is ∼0.6 mm long, this equals to an increase in axonal conduction velocity from 1.15 ± 0.09 m/s at 1hPE to 2.07 ± 0.21 m/s in 24PE flies (Fig. 1*A*,*Civ*; Table 2). We next aimed at addressing the ionic basis of this postnatal increase in axonal conduction velocity of the GF interneuron.

**Table 1.** Latency measurements in the GF-TTM pathways, in the TTMn-TTM Sub-pathways, and in the GF axon at 1hPE (gray) and 24hPE (black) control as compared to 1hPE and 24hPE in flies where genes encoding ion channels were expressed or knocked down specifically in the GF interneurons

	GF-TTM; mean ± SEM (*n*)		TTMn-TTM; mean ± SEM (*n*)		GF=(GF-TTM)-(TTMn-TTM); mean ± SEM (*n*)	
Genotype	1hPE	24hPE	1hPE	24hPE	1hPE	24hPE
Control	1.32 ± 0.02 ms (7)	1.13 ± 0.02 ms (9)	0.80 ± 0.04 ms (5)	0.84 ± 0.02 ms (8)	0.52 ± 0.04 ms (7)	0.29 ± 0.03 ms (9)
Para-RNAi	1.52 ± 0.03 ms (8)	1.29 ± 0.03 ms (8)	0.83 ± 0.03 ms (5)	0.82 ± 0.03 ms (5)	0.69 ± 0.04 ms (8)	0.47 ± 0.03 ms (8)
NaChBac	1.21 ± 0.02 ms (9)	1.02 ± 0.01 ms (8)	0.86 ± 0.02 ms (6)	0.86 ± 0.02 ms (7)	0.35 ± 0.02 ms (9)	0.16 ± 0.03 ms (8)
DmCa1D-RNAi	1.59 ± 0.03 ms (9)	1.48 ± 0.02 ms (9)	0.86 ± 0.02 ms (5)	0.84 ± 0.02 ms (6)	0.73 ± 0.05 ms (9)	0.64 ± 0.04 ms (9)
Sh-RNAi	1.43 ± 0.04 ms (8)	1.25 ± 0.02 ms (8)	0.87 ± 0.03 ms (5)	0.84 ± 0.03 ms (5)	0.56 ± 0.05 ms (8)	0.41 ± 0.04 ms (8)
Shal-RNAi	1.42 ± 0.03 ms (9)	1.17 ± 0.02 ms (8)	0.87 ± 0.02 ms (6)	0.84 ± 0.03 ms (7)	0.55 ± 0.03 ms (9)	0.33 ± 0.03 ms (8)
Slo-RNAi	1.58 ± 0.04 ms (9)	1.31 ± 0.02 ms (10)	0.87 ± 0.01 ms (5)	0.86 ± 0.02 ms (7)	0.71 ± 0.04 ms (9)	0.45 ± 0.03 ms (10)

The latency in the GF axon is estimated by subtracting the TTMn-TTM latency from the GF-TTM latency. Data are shown as means ± SEM; *n*, number of preparation tested.

**Table 2.** % Increase (+) or decrease (–) in GF axonal conduction speed due to targeted expression or knock-down of genes encoding ion channels in the GF interneurons at 1hPE (gray) and 24hPE (black) flies, or during the first day of the fly life (1–24hPE)

	% Change due to “knock-down” or “expression” of genes encoding ionic channels		% Change due to postnatal maturation
Genotype	1hPE	24hPE	1–24hPE
Control	-------	------	80%***
Para-RNAi	–32%*	–62%***	47%***
NaChBac	+49%***	+81%**	119%****
DmCa1D-RNAi	–40%**	–120%****	15%; ns
Sh-RNAi	–7%; ns	–42%*	36%***
Shal-RNAi	–6%; ns	–14%; ns	67%***
Slo-RNAi	–35%**	–56%**	56%****

Asterisks indicate *p* values from one-way ANOVA with *post hoc* Dunnett’s tests (*p < 0.05, **p < 0.01, ***p < 0.001, ****p < 0.0001, n.s., *p* > 0.05).

### Voltage-gated sodium and L-type calcium channels contribute to the GF conduction velocity increase postnatally

**Figure 2.**
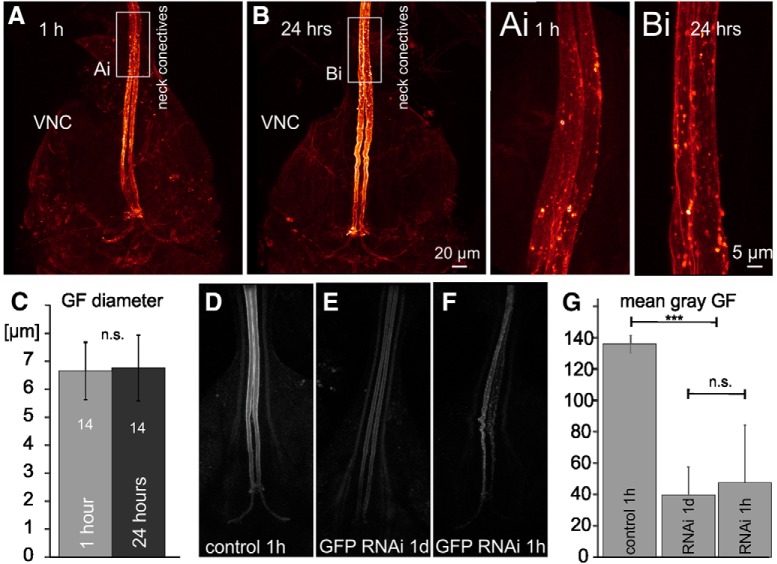
The GF axon diameter does not increase postnatally, and GFP-RNAi decreases GFP fluorescence similarly at 1 h and 24 h post-eclosion. ***A***, Representative confocal image stacks of the GF interneuron axons between the neck connectives and the terminals in the VNC at 1hPE (***A***) and at 24hPE (***B***). To avoid potential histology artifacts images were taken live in saline with a 60× water dipping lens from freshly dissected animals with UAS-cd4-tomato expression in the GF. White boxes indicate areas shown as selective enlargements in ***Ai***, ***Bi***. ***C***, Quantification from 14 axons at each stage shows that GF axon diameter is similar at 1hPE and 24hPE (*p* > 0.6, Student’s *t* test). VNC, ventral nerve cord. ***D–G***, In comparison to control (***D***), targeted GFP RNAi knock-down reduces GFP fluorescence at both stages tested, 24hPE (***E***) and 1hPE (***F***). ***G***, Quantification of mean gray levels in eight bit tiff images of the GF axon (0–254 gray levels) reveals a significant reduction by ∼70% but no differences between both stages tested (ANOVA with Newman–Keuls *post hoc* testing); ***p < 0.001; n.s., *p* > 0.3.

Potential mechanisms underlying postnatal increases in axonal conduction velocity are (1) an enlargement in axon diameter (this would result in decreased axial resistance, thus increasing the length constant λ; Hodgkin, 1954); (2) improving glial wrapping (this would result in increased membrane resistance, thus increasing λ; Moore et al., 1978); and (3) hypothetically, changes in the complement, properties, and/or densities of ion channels located in the axonal membrane. First, axon diameter changes can be ruled unlikely because it has been suggested that the axon of the GF reaches its final diameter before eclosion (Allen et al., 1998). Indeed, confocal images demonstrate that the GF outer axon diameter (∼7 μm) is not different between 1hPE and 24hPE flies (Fig. 2). But note that CLSM just uncovers the outer axon diameter. EM would be required to rule out small changes in the effective inner diameter that determines axoplasmatic resistance. However, we feel that subtle differences in effective axon diameter that might be hidden to CLSM diameter measurements are difficult to reconcile with the observed 80% increase in conduction velocity. Second, the GF is a non-myelinated invertebrate axon, thus making major effects of glial insulation on AP conduction speed unlikely. Please note that GF axonal conduction velocity is roughly similar to that of unmyelinated C-fibers in vertebrates (see Discussion). Therefore, we hypothesized that postnatal changes in the expression of axonally localized voltage-gated ion channels may underlie postnatal AP conduction velocity increases in the GF.

To test this, we manipulated the expression levels of selected ion channels specifically in the GF by targeting UAS-transgenes with a *split-GAL4* driver that expresses solely in the GF interneurons (von Reyn et al., 2014), and compared axonal conduction velocity at 1 h (1hPE) and at 24 h (24hPE) post adult eclosion. This approach ideally requires identical levels of RNAi knock-down efficacy in the GF interneuron at both stages. To exclude largely different expression levels of Gal4 at both staged, we estimated knock-down efficacy in the GF by expressing UAS-RNAi for GFP under the control of the GF selective *split-GAL4;UAS-mcd8-GFP* driver and compared fluorescence intensity at both stages (Fig. 2*D–G*). At 1hPE, targeted expression of UAS-GFP-RNAi decreased mean GFP fluorescence intensity in the GF interneuron highly significantly (Fig. 2*D*,*F*) by ∼70% (Fig. 2*G*), thus demonstrating significant effects of RNAi at 1hPE. GFP fluorescence was not further decreased at 24hPE (Fig. 2*D*,*F*,*G*). Although this approach provides only a rough estimate of RNAi knock-down efficacy, and generalization for different RNAi constructs is not readily possible, these data indicate that UAS-RNAi expression in the GF may provide roughly similar knock-down efficacy at 1hPE and at 24hPE. However, this does not exclude different knock-down efficacies of UAS-RNAi constructs for different ion channels. Four of the five UAS-RNAi constructs for different ion channels used in this study (see below) have previously been confirmed to cause 50–70% knock-down efficacy in larval *Drosophila* motoneurons by means of electrophysiology and/or immunocytochemistry (see Materials and Methods). Taken together, it seems plausible to assume that each RNAi constructs used caused knock-down of the respective channels in the GF neuron, and that for a given ion channel knock-down efficacy was similar at both stages tested.

**Figure 3.**
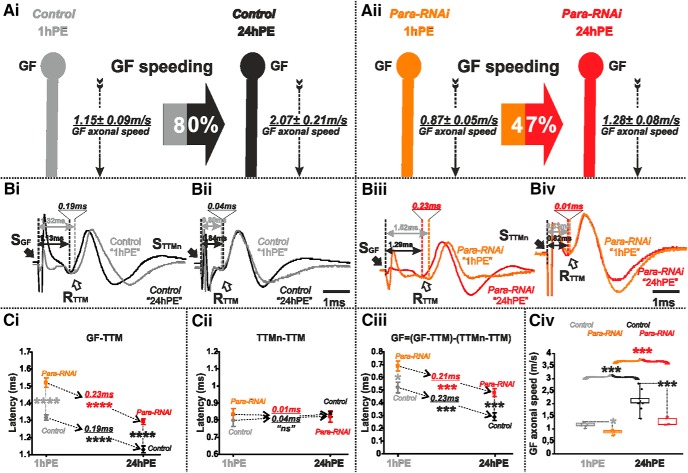
*Para*-RNAi decreases postnatal conduction velocity speeding in the GF. ***A***, GF schematic depiction at 1hPE (gray) and 24hPE (black) in controls (***Ai***) as compared to 1hPE (orange) and 24hPE (red) in flies with *para* RNAi (***Aii***). GF axonal conduction velocity (dotted arrow) is given for each experimental group. The percentage of the GF postnatal speeding in control (gray-black arrow, 80%) is strongly reduced by *para* RNAi knock-down (orange-red arrow, 47%). ***B***, Representative TTM muscle APs recorded after GF (***Bi***) or TTMn (***Bii***) stimulation at 1hPE (gray) and at 24hPE (black) in control flies as compared to *para* RNAi knock-down (***Biii***, GF stimulation; ***Biv***, TTMn stimulation) at 1hPE (orange) and at 24hPE (red). Times above double arrows and between dotted lines indicate the latency between GF (or TTMn) and TTM. Latency differences between stages are underlined for control and for *para* RNAi knock-down. ***C,*** Latency measurements in the GF-TTM pathway (***Ci***), in the TTMn-TTM sub-pathway (***Cii***), in the GF axon (***Ciii***) and measurements of the GF axonal speed (**C*iv***), at 1hPE (gray) and 24hPE (black) in controls and at 1hPE (orange) and 24hPE (red) in flies expressing *para* RNAi transgene. Underlined times between dotted arrows indicate the latency difference between the two stages in control and *para* RNAi knock-down. Data are shown as means ± SEM (***Ci–Ciii***). Dots on box plots showcase the measurements from individual flies (***Civ***). Asterisks indicate *p* values from one-way ANOVA with *post hoc* Dunnett’s tests (*p < 0.05, ***p < 0.001, ****p < 0.0001, n.s., *p* > 0.29).

We first tested the contribution of voltage-gated sodium channels for axonal conduction velocity. Fast sodium channels are required for AP generation and propagation in the GF (Tanouye et al., 1981; Tanouye and Ferrus, 1985; von Reyn et al., 2014). The only gene encoding fast voltage-gated sodium channels in *Drosophila* is *paralytic* (*para)* or *DmNa_v_* (Feng et al., 1995; Mee et al., 2004). We lowered the amount of *para* expression in the GF interneuron by targeted expression of *para*-RNAi. For both stages tested (1hPE and 24hPE), this resulted in a significant decrease in axonal conduction velocity in the GF interneuron (Fig. 3*A*,*B*), which was calculated from measurements of the response latency increases in the GF-TTM pathway with and without para RNAi (Fig. 3*Bi*,*Biii*,*Ci*). The latency between TTMn and TTM was not affected because the RNAi knock-down was targeted to the GF only (Fig. 3*Bii*,*Biv*,*Cii*). Keeping in mind that the GF axonal conduction time equals the time difference between the GF-TTM and the TTMn-TTM latencies, *para* RNAi knock-down in the GF increases axonal conduction time from 0.52 ± 0.04 to 0.69 ± 0.04 ms at 1hPE and from 0.29 ± 0.03 to 0.47 ± 0.03 ms at 24hPE (Fig. 3*Ciii*; Table 1). This corresponds to a 32% decrease in GF axonal conduction velocity at 1hPE (1.15 ± 0.09 m/s in control to 0.87 ± 0.05 m/s in *para* RNAi knock-down) but in a 62% decrease in conduction velocity at 24hPE (from 2.07 ± 0.21 m/s in control to 1.28 ± 0.08 m/s in *para* RNAi knock-down; Fig. 3*A*,*Civ*; Table 2). Therefore, *para* RNAi had a stronger effect in more mature GF interneurons. However, even in the presence of *para* RNAi we found a postnatal decrease in GF conduction time of 0.21 ms (from 0.69 ± 0.04 to 0.47 ± 0.03 ms; Fig. 3*Ciii*; Table 1), corresponding to a postnatal speeding of only 47% (Fig. 3*Aii*; Table 2), roughly half the postnatal speeding found in control (80%; Figs. 3*Ai*; Table 2).

**Figure 4.**
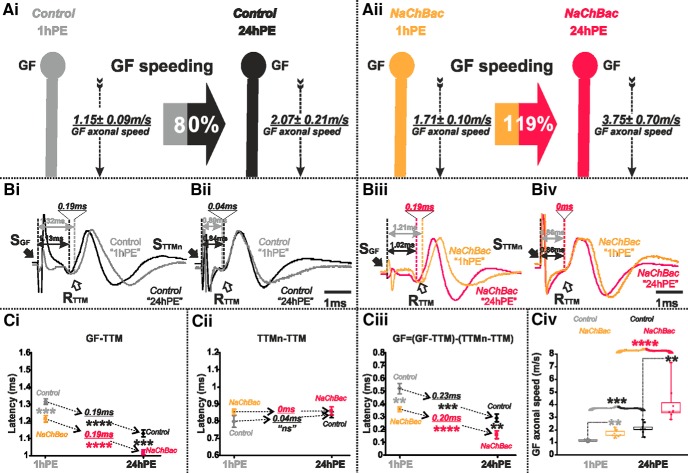
Expression of extra sodium channels (NaChBac) increases postnatal conduction velocity speeding in the GF. ***A***, GF schematic depiction at 1hPE (gray) and 24hPE (black) in controls (***Ai***) as compared to 1hPE (light orange) and 24hPE (neon red) in flies expressing NaChBac channels (***Aii***). GF axonal conduction velocity (dotted arrow) is given for each experimental group. The percentage of the GF postnatal speeding in control (gray-black arrow, 80%) is strongly increased by NaChBac channels expression (light orange-neon red, 119%). ***B***, Representative TTM muscle APs recorded after GF (***Bi***) or TTMn (***Bii***) stimulation at 1hPE (gray) and at 24hPE (black) in control flies as compared to flies expressing NaChBac channels (***Biii***, GF stimulation; ***Biv***, TTMn stimulation) at 1hPE (light orange) and at 24hPE (neon red). Times above double arrows and between dotted lines indicate the latency between GF (or TTMn) and TTM. Latency differences between stages are underlined for control and for NaChBac channels expression. ***C,*** Latency measurements in the GF-TTM pathway (***Ci***), in the TTMn-TTM sub-pathway (***Cii***), in the GF axon (***Ciii***) and measurements of the GF axonal speed (***Civ***), at 1hPE (gray) and 24hPE (black) in controls and at 1hPE (light orange) and 24hPE (neon red) in flies expressing *NaChBac* transgene. Underlined times between dotted arrows indicate the latency difference between the two stages in control and flies expressing NaChBac sodium channels. Data are shown as means ± SEM (***Ci–Ciii***). Dots on box plots showcase the measurements from individual flies (***Civ***). Asterisks indicate *p* values from one-way ANOVA with *post hoc* Dunnett’s tests (**p < 0.01, ***p < 0.001, ****p < 0.0001, n.s., *p* > 0.15).

Since GF AP conduction velocity was decreased on lowering the amount of Para channels, we hypothesized that conversely, an upregulation of voltage-gated sodium channel expression will increase GF axonal conduction velocity. Although *Drosophila* Para channels have been functionally characterized in *Xenopus* oocytes (Lin et al., 2009), UAS-*para* transgenes have so far not been successfully expressed in flies (Lin and Baines, 2015). Therefore, we drove expression of a transgene encoding the bacterial sodium channels, NaChBac, specifically in the GF (GF *split-Gal4*). Functional sodium current through NaChBac expressed in *Drosophila* neurons and muscles has previously been demonstrated (Luan et al., 2006; Nitabach et al., 2006). We predicted that an increase in the total number of (bacterial + native) sodium channels will increase axonal conduction velocity. Indeed, for both stages tested (1hPE and 24hPE), the latency in the GF-TTM pathway was significantly lower in flies expressing the *NaChBac* transgene than in control ones (Fig. 4*Bi*,*Biii*,*Ci*). Again, given that expression was restricted to the GF interneuron, the latency between TTMn and TTM remained unchanged following *NaChBac* transgene expression (Fig. 4*Bii*,*Biv*,*Cii*). Thus, in contrast to the increased axonal conduction time on *para* RNAi knock-down, the expression of extra sodium channels significantly decreased conduction time in the GF axon of 1hPE flies (0.52 ± 0.04 vs 0.35 ± 0.02 ms) and of 24hPE flies (0.29 ± 0.03 vs 0.16 ± 0.03 ms; Fig. 4*Ciii*; Table 1), and therefore, increased GF conduction speed (1.15 ± 0.09 vs 1.71 ± 0.10 m/s) at 1hPE and (2.07 ± 0.21 vs 3.75 ± 0.70 m/s) at 24hPE (Fig. 4*A*,*Civ*). Therefore, with extra sodium (NaChBac) channel expression, the postnatal increase in AP conduction velocity is further enhanced to 119% (Fig. 4*Aii*; Table 2), as compared to the 80% enhancement in control (Figs. 4*Ai*, 8; Table 2). To sum up, sodium channels control axonal conduction speed in the GF and may contribute to postnatal maturation.

**Figure 5.**
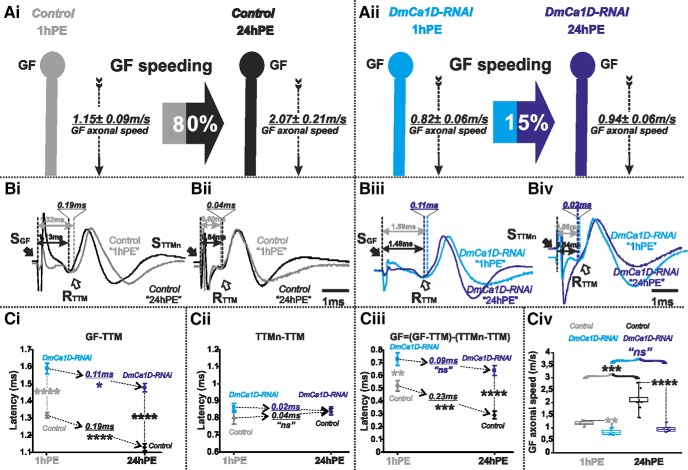
*DmCa1D*-RNAi almost eliminates postnatal conduction velocity speeding in the GF. ***A***, GF schematic depiction at 1hPE (gray) and 24hPE (black) in controls (***Ai***) as compared to 1hPE (cyan) and 24hPE (blue) in flies with *DmCa1D* RNAi (***Aii***). GF axonal conduction velocity (dotted arrow) is given for each experimental group. The percentage of the GF postnatal speeding in control (gray-black arrow, 80%) is extremely reduced by *DmCa1D* RNAi knock-down (cyan-blue arrow, 15%). ***B***, Representative TTM muscle APs recorded after GF (***Bi***) or TTMn (***Bii***) stimulation at 1hPE (gray) and at 24hPE (black) in control flies as compared to *DmCa1D* RNAi knock-down (***Biii***, GF stimulation; ***Biv***, TTMn stimulation) at 1hPE (cyan) and at 24hPE (blue). Times above double arrows and between dotted lines indicate the latency between GF (or TTMn) and TTM. Latency differences between stages are underlined for control and for *DmCa1D* RNAi knock-down. ***C,*** Latency measurements in the GF-TTM pathway (***Ci***), in the TTMn-TTM sub-pathway (***Cii***), in the GF axon (***Ciii***) and measurements of the GF axonal speed (***Civ***), at 1hPE (gray) and 24hPE (black) in controls and at 1hPE (cyan) and 24hPE (blue) in flies expressing *DmCa1D* RNAi transgene. Underlined time values between dotted arrows indicate the latency difference between the two stages in control and *DmCa1D* RNAi knock-down. Data are shown as means ± SEM (***Ci–Ciii***). Dots on box plots showcase the measurements from individual flies (***Civ***). Asterisks indicate *p* values from one-way ANOVA with *post hoc* Dunnett’s tests (*p < 0.05, **p < 0.01, ***p < 0.001, ****p < 0.0001, n.s., *p* > 0.05).

We next tested possible contributions of other voltage-gated ion channels localized in the axon. It has recently been demonstrated that L-type calcium channels encoded by Dmca1D localize to motoneuron axons, where they augment high frequency firing (Kadas et al., 2017). Targeted *DmCa1D* RNAi knock-down, specifically in the GF, dramatically increased response latency in the GF-TTM pathway at both stages examined (Fig. 5*Bi*,*Biii*,*Ci*). Given that the TTMn and its neuromuscular junction with TTM (TTMn/TTM) do not contribute to that change in response latency (Fig. 5*Bii*,*Biv*,*Cii*), the data demonstrate that *DmCa1D* RNAi knock-down significantly increases GF axonal conduction time, both at 1hPE (0.52 ± 0.04 vs 0.73 ± 0.05 ms) and at 24hPE (0.29 ± 0.03 vs 0.64 ± 0.04 ms; Fig. 5*Ciii*; Table 1). Noticeably, this corresponds to a decrease in GF axonal conduction speed of 40% (1.15 ± 0.09 vs 0.82 ± 0.06 m/s) at 1hPE and a much higher decrease of 120% (2.07 ± 0.21 vs 0.94 ± 0.06 m/s) at 24hPE (Fig. 5*A*,*Civ*; Table 2). Consequently, in *DmCa1D* RNAi knock-down flies postnatal speeding of GF axonal conduction velocity was reduced to just 15% (Fig. *5Aii*; Table 2), as compared to 80% in control (Fig. 4*Ai*, 8; Table 2). Hence, in addition to reducing conduction speed at both stages tested, *DmCa1D* plays a major role in postnatal conduction velocity speeding.

### Shaker and Slowpoke potassium channels control the GF conduction velocity but only Shaker contributes to its postnatal increment

In addition to having identified two voltage-gated channels mediating inward current that increase AP conduction velocity, we also tested for potential roles of potassium outward currents. We tested the *Drosophila* homologs of the mammalian K_V_1 and K_V_4 voltage-gated potassium channels, such as Shaker and Shal, and the BK channel homolog Slowpoke. In *Drosophila* motoneurons, Shaker and Shal mediate fast activating, fast inactivating voltage-gated potassium currents (Ryglewski and Duch, 2009), while Slowpoke underlies transient calcium-activated potassium currents (Kadas et al., 2015). Expression of each of these potassium channels was reduced by selective expression of the respective UAS-RNAi transgenes specifically in the GF interneuron (von Reyn et al., 2014).

**Figure 6.**
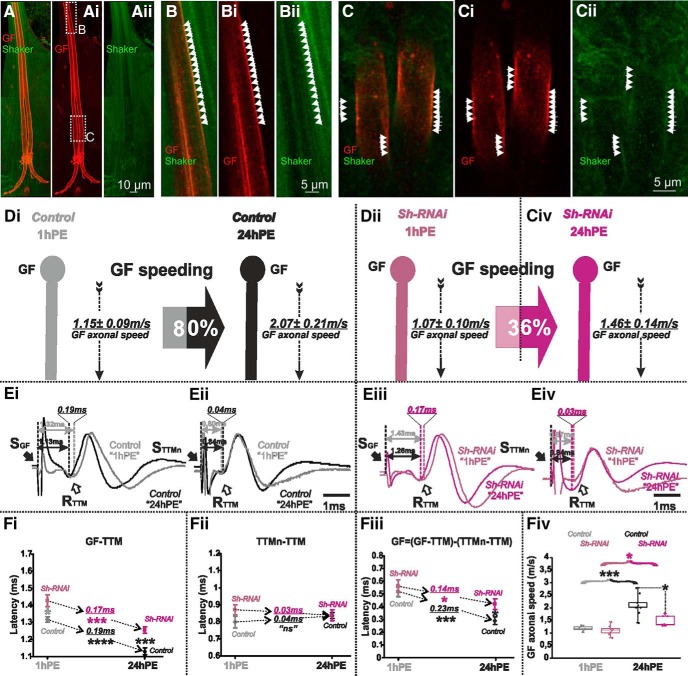
Shaker channels are localized in the GF axon and *Sh*-RNAi decreases postnatal conduction velocity speeding. ***A–Aii***, Projection views of a representative confocal image stack from the GF with UAS-cd4-tomato expression (red) and Shaker channel expression as visualized by an endogenous GFP tag (green). Dotted white boxes in ***Ai*** indicate the areas which are selectively enlarged in ***B***, ***C*** and shown as z-projections of 1 µm (three optical sections). White arrowheads demark areas with overlap of patches of GF axonal membrane (red) and Shaker-GFP label. The GF axonal lumen is mostly devoid of Shaker-GFP label. ***D***, GF schematic depiction at 1hPE (gray) and 24hPE (black) in controls (***Di***) as compared to 1hPE (violet) and 24hPE (purple) in flies with *Sh* RNAi (***Dii***). GF axonal conduction velocity (dotted arrow) is given for each experimental group. The percentage of the GF postnatal speeding in control (gray-black arrow, 80%) is strongly reduced by *Sh* RNAi knock-down (violet-purple arrow, 36%). ***E***, Representative TTM muscle APs recorded after GF (***Ei***) or TTMn (***Eii***) stimulation at 1hPE (gray) and at 24hPE (black) in control flies as compared to *Sh* RNAi knock-down (***Eiii***, GF stimulation; ***Eiv***, TTMn stimulation) at 1hPE (violet) and at 24hPE (purple). Times above double arrows and between dotted lines indicate the latency between GF (or TTMn) and TTM. Latency differences between stages are underlined for control and for *Sh* RNAi knock-down. ***F***, Latency measurements in the GF-TTM pathway (***Fi***), in the TTMn-TTM sub-pathway (***Fii***), in the GF axon (***Fiii***), and measurements of the GF axonal speed (***Fiv***), at 1hPE (gray) and 24hPE (black) in controls and at 1hPE (violet) and 24hPE (purple) in flies expressing *Sh* RNAi. Underlined times between dotted arrows indicate the latency difference between the two stages in control and *Sh* RNAi knock-down. Data are shown as means ± SEM (***Fi–Fiii***). Dots on box plots showcase the measurements from individual flies (***Fiv***). Asterisks indicate *p* values from one-way ANOVA with *post hoc* Dunnett’s tests (*p < 0.05, ***p < 0.001, ****p < 0.0001, n.s., *p* > 0.15).

Axonal localization of Shaker potassium channel in *Drosophila* DLM flight MNs has previously been demonstrated by immunocytochemistry (Ryglewski and Duch, 2009). Although light microscopy lacks sufficient spatial resolution to unambiguously assign labeled ion channel proteins to the membrane of an identified neuron *in situ*, high resolution confocal laser microscopy of UAS-tomato expressing GF axons in animals with endogenously GFP-tagged Shaker channels suggested that Shaker channels likely localize to the GF axonal membrane (Fig. 6*A–C*). Representative projection views show the GF axons between the cervical connectives and their axon terminals in the thoracic neuromere, as well as many additional Shaker positive descending axons (Fig. 6*A*). Single optical sections (0.3-µm section thickness) reveal patches of GFP tagged Shaker channels that co-localize with segments of UAS-tomato positive GF axonal membrane (Fig. 6*B*,*C*), whereas the lumen of the large diameter GF axons is mostly devoid of Shaker label. This indicated functional Shaker channel localization to the GF axonal membrane.

Targeted RNAi knock-down of *shaker* (*sh*) in the GF significantly increased the latency in the GF-TTM pathway at both stages, although the effect is smaller at 1hPE (Fig. 6*Ei*,*Eiii*,*Fi*). Again, motoneuron axonal conduction and neuromuscular transmission were not significantly affected by the expression of *sh* RNAi in the GF interneuron (Fig. 6*Eii*,*Eiv*,*Fii*). *Sh* RNAi knock-down in the GF of 1hPE flies did not cause a statistically significant change in the GF conduction time (0.56 ± 0.05 vs 0.52 ± 0.04 ms of control; Fig. 6*Fiii*; Table 1). By contrast, *sh* RNAi knock-down in the GF of 24hPE flies significantly enhanced the GF conduction time (0.29 ± 0.03 vs 0.41 ± 0.04 ms of controls; Fig. 6*Fiii*; Table 1). Consequently, *sh* RNAi knock-down decreased GF axonal conduction speed significantly at 24hPE (2.07 ± 0.21 vs 1.46 ± 0.14 m/s), but not at 1hPE (1.15 ± 0.09 vs 1.07 ± 0.10 m/s; Fig. 6*A*,*Fiv*). Given that *sh* RNAi knock-down reduced GF conduction at 24hPE by 42%, but only by 7% at 1hPE (Table 2), postnatal conduction speeding was only 36% (Fig. 6*Dii*; Table 2), as compared to 80% in control (Fig. 6*Di*; Table 2).

**Figure 7.**
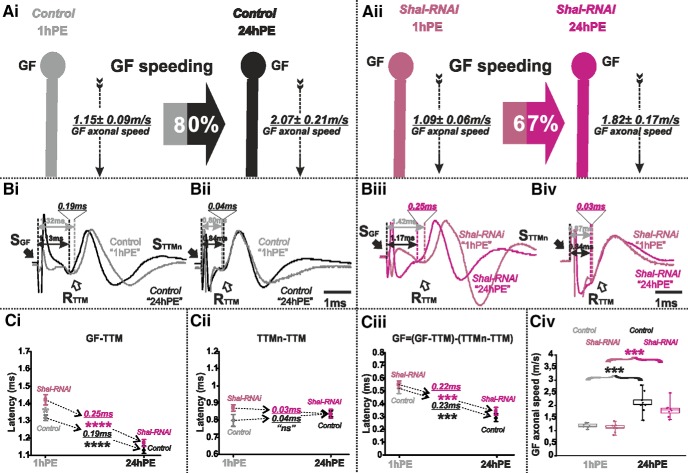
*Shal*-RNAi does not affect postnatal conduction velocity speeding in the GF. ***A***, GF schematic depiction at 1hPE (gray) and 24hPE (black) in controls (***Ai***) as compared to 1hPE (violet) and 24hPE (purple) in flies with *Shal* RNAi (***Aii***). GF axonal conduction velocity (dotted arrow) is given for each experimental group. The percentage of the GF postnatal speeding in control (gray-black arrow, 80%) is not significantly reduced by *Shal* RNAi knock-down (violet-purple arrow, 67%). ***B***, Representative TTM muscle APs recorded after GF (***Bi***) or TTMn (***Bii***) stimulation at 1hPE (gray) and at 24hPE (black) in control flies as compared to *Shal* RNAi knock-down (***Biii***, GF stimulation; ***Biv***, TTMn stimulation) at 1hPE (violet) and at 24hPE (purple). Times above double arrows and between dotted lines indicate the latency between GF (or TTMn) and TTM. Latency differences between stages are underlined for control and for *Shal* RNAi knock-down. ***C***, Latency measurements in the GF-TTM pathway (***Ci***), in the TTMn-TTM sub-pathway (***Cii***), in the GF axon (***Ciii***) and measurements of the GF axonal speed (***Civ***), at 1hPE (gray) and 24hPE (black) in controls and at 1hPE (violet) and 24hPE (purple) in flies expressing *Shal* RNAi transgene. Underlined time values between dotted arrows indicate the latency difference between the two stages in control and *Shal* RNAi knock-down. Data are shown as means ± SEM (***Ci–Ciii***). Dots on box plots showcase the measurements from individual flies (***Civ***). Asterisks indicate *p* values from one-way ANOVA with *post hoc* Dunnett’s tests (*p < 0.05, ***p < 0.001, ****p < 0.0001, n.s., *p* > 0.09).

Targeted RNAi knock-down of *shal* in the GF caused a small but statistically significant increase of the latency recorded in the GF-TTM pathway at 1hPE (Fig. 7*Bi*,*Biii*,*Ci*), without affecting the latency between TTMn and TTM (Fig. 7*Bii*,*Biv*,*Cii*). By contrast, it did not significantly affect response latency of the GF-TTM pathway at 24hPE (Fig. 7*Bi*,*Biii*,*Ci*). Similar to *sh* RNAi knock-down, the almost negligible increase in response latency in the GF-TTM pathway at 1hPE did not account for a statistically significant increase in the GF conduction time (Fig. 7*Ciii*). Indeed, *shal* RNAi knock-down had only small effects on GF conduction velocity, a 6% at decreases at 1hPE (1.15 ± 0.09 vs 1.09 ± 0.06 m/s) and a 14% decrease at 24hPE (2.07 ± 0.21 vs 1.82 ± 0.17 m/s; Fig. 7*A*,*Civ*; Table 2). Therefore, Shal RNAi knock-down lowered postnatal speeding in GF conduction velocity only minimally, from 80% in controls (Figs. 7*Ai*; Table 2) to 67% (Fig. 7*Aii*; Table 2).

**Figure 8.**
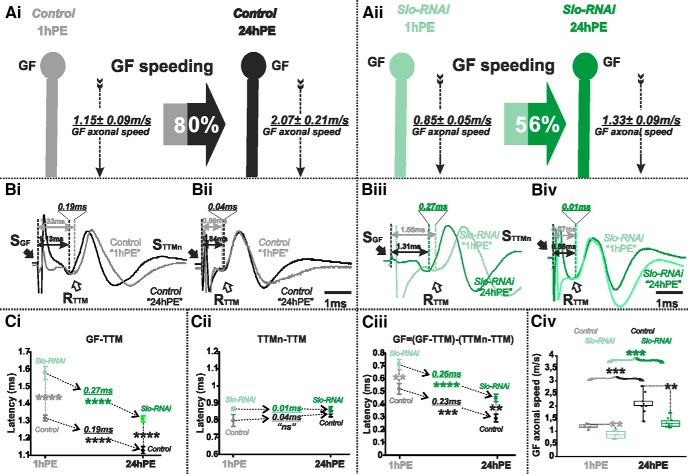
*Slo*-RNAi affects slightly postnatal conduction velocity speeding in the GF. ***A***, GF schematic depiction at 1hPE (gray) and 24hPE (black) in controls (***Ai***) as compared to 1hPE (mint green) and 24hPE (green) in flies with *Slo* RNAi (***Aii***). GF axonal conduction velocity (dotted arrow) is given for each experimental group. The percentage of the GF postnatal speeding in control (gray-black arrow, 80%) is moderately reduced by *Slo* RNAi knock-down (mint green-green arrow, 56%). ***B***, Representative TTM muscle APs recorded after GF (***Bi***) or TTMn (***Bii***) stimulation at 1hPE (gray) and at 24hPE (black) in control flies as compared to *Slo* RNAi knock-down (***Biii***, GF stimulation; ***Biv***, TTMn stimulation) at 1hPE (mint green) and at 24hPE (green). Times above double arrows and between dotted lines indicate the latency between GF (or TTMn) and TTM. Latency differences between stages are underlined for control and for *Slo* RNAi knock-down. ***C***, Latency measurements in the GF-TTM pathway (***Ci***), in the TTMn-TTM sub-pathway (***Cii***), in the GF axon (***Ciii***) and measurements of the GF axonal speed (***Civ***), at 1hPE (gray) and 24hPE (black) in controls and at 1hPE (mint green) and 24hPE (green) in flies expressing *Slo* RNAi transgene. Underlined time values between dotted arrows indicate the latency difference between the two stages in control and *Slo* RNAi knock-down. Data are shown as means ± SEM (***Ci–Ciii***). Dots on box plots showcase the measurements from individual flies (***Civ***). Asterisks indicate *p* values from one-way ANOVA with *post hoc* Dunnett’s tests (**p < 0.01, ***p < 0.001, ****p < 0.0001, n.s., *p* > 0.1).

Finally, RNAi knock-down of *slowpoke* (or *slo*) significantly increased GF-TTM pathway latency (Fig. 8*Bi*,*Biii*,*Ci*) and axonal conduction time in the GF (Fig. 8*Ciii*) at both stages tested (1hPE and 24hPE). Again, TTMn to TTM was not affected (Fig. 8*Bii*,*Biv*,*Cii*) by *slo* RNAi knock-down in the GF. Given that *slo* RNAi exerted a slightly stronger effect at 24hPE than at 1hPE (56% decrease in the GF conduction speed at 24hPE vs 35% at 1hPE; Table 2), the postnatal speeding of the GF conduction velocity in the presence of *slo* RNAi knock-down was reduced from 80% in control to 56% with *slo* RNAi expression (Fig. 8*A*,*Civ*; Table 2).

In summary, Shaker voltage-gated and Slowpoke calcium-activated potassium channels significantly increase axonal conduction velocity in the GF. However, in the presence of constant RNAi expression, postnatal speeding of the GF axonal conduction is affected much stronger with *sh* RNAi than with *slo* RNAi (see Fig. 6*A* vs Fig. 8*A*). Knock-down of *para* reduces postnatal speeding to a similar amount as *sh* RNAi (see Fig. 3*A* vs Fig. 6*A*). By contrast, targeted expression of *Dmca1D* RNAi eliminates postnatal speeding of axonal conduction velocity almost completely (Fig. 5*A*). In sum, postnatal upregulation of both Shaker outward and sodium inward current likely contributes to the postnatal increase of the GF axonal conduction speed, but up-regulation of L-type calcium channels seems most important.

## Discussion

### AP conduction velocity in the GF interneuron increases postnatally

We show that information transfer through the *Drosophila* GFS becomes significantly faster during the first 24 h of adult life. We ruled out functional changes in the PSI and/or the PSI/MN synapse, because these contribute only in the GF-DLM pathway, but both GFS branches showed similar decreases in response latency. This is in accord with previous findings that electrical (GF to TTMn, GF to PSI; Phelan et al., 1996; Jacobs et al., 2000) and chemical (PSI to MN1–5; Allen et al., 2006) synapse formation are completed during pupal stages. We also ruled out postnatal changes of MNs or neuromuscular synapses, because muscle response latencies to MN firing remained unaltered, and MN axons expand over the developing DLM muscles already by ∼10 h APF (Fernandes and Keshishian, 1996, 1998; Consoulas et al., 2002). Consequently, decreased response latency is caused by an 80% increase in GF axonal conduction velocity from 1.15 ± 0.09 m/s at 1hPE to 2.07 ± 0.21 m/s at 24PE. Although AP travel time through the GF axon is decreased by only 0.2 ms, at the scale of a fruit fly this might be relevant. For a threat approaching with ∼30 km/h (roughly the speed of a frog tongue) 0.2 ms provide an advantage of 1.6 mm, more than half a fly’s body length.

Axonal conduction velocity can be increased by enlarging axon diameter, improving plasma membrane insulation by myelination (Fitzgerald, 1985; Fulton, 1987; Hartline and Colman, 2007), or by adjusting ion channel expression levels, or by modifying channel properties (see below). First, although glial wrapping may affect conduction velocity in unmyelinated invertebrate axons (Dutta et al., 2016), we judge this mechanism unlikely, because mature GF conduction velocity is ∼2 m/s, comparable to vertebrate unmyelinated C-fibers. Second, GF morphologic maturation is reportedly completed during pupal life (Allen et al., 1998), and we experimentally ruled out postnatal increases in outer axon diameter by CLSM. Therefore, we hypothesized changes in ion channel expression or in ion channel function to underlie postnatal conduction velocity increases. Postnatal changes in sodium and potassium currents are reported in rat optic nerve (Foster et al., 1982). In trigeminal ganglion sensory neuron (Aδ) postnatal conduction velocity increases are accompanied by a sharpening of AP shape (Cabanes et al., 2002). GF APs depend on sodium inward and potassium currents (Tanouye et al., 1981; Tanouye and Ferrus, 1985; von Reyn et al., 2014). Therefore, we first tested the role of fast sodium channels.

### Increased sodium channel expression is required for GF postnatal speeding

Moderate RNAi knock-down of *para* (*DmNa_v_*) decreased GF axonal conduction velocity. Similarly, in unmyelinated axons innervating the rat cranial meninges, reduced extracellular sodium concentrations or low doses of sodium channel blockers decrease conduction velocity (De Col et al., 2008). By contrast, overexpressing of bacterial sodium channels (NaChBac) increased GF axonal conduction velocity. Since voltage-gated sodium channels mediate the rising phase of the AP via positive feedback (Catterall, 2000), increased channel numbers likely accelerate depolarization speed. Conversely, a faster depolarization may accelerate sodium channel inactivation and thus increase repolarization speed.

Reducing sodium channel expression affected GF conduction velocity in mature flies significantly stronger than in newly eclosed ones. Therefore, the GF likely becomes equipped with a larger number of fast sodium channels during the first day of adult life. Alternatively, axonal conductance velocity could be regulated by the expression of different sodium channel isoforms. A variety of Para splice variants with different activation/inactivation kinetics exist (Olson et al., 2008; Lin et al., 2009), and sodium current amplitude is affected by activity dependent regulation of *para* mRNA levels and translation (Baines et al., 2001; Baines, 2003; Mee et al., 2004; Muraro et al., 2008).

Similarly, in mammals, the ten genes (*SCN1A-SCN11A*; Goldin, 2001) encoding sodium channel α-subunits show differential expression patterns during postnatal maturation. Although it is known that the expression of TTX sensitive, fast Na_v_1.6 and Na_v_1.7 channels increases postnatally (Felts et al., 1997), while the expression of TTX resistant, slower kinetics Na_v_1.8 and Na_v_1.9 channels peaks earlier (Benn et al., 2001), a direct link to postnatal adjustments of conduction velocities has not been made. In rat DRG, TTX resistant sodium channels may contribute to the slow conduction velocity of unmyelinated C-fibers (Ogata and Tatebayashi, 1992).

Therefore, especially in non-myelinated axons, the regulation of ion channel expression seems to provide an effective means to adjust conduction velocity. However, despite our finding that overexpression of bacterial sodium channels increased conduction velocity, whereas reduction of para transcript by RNAi reduced conduction velocity, we cannot rule that normal postnatal speeding may be caused by additional mechanisms, such as the expression of accessory subunits, differential splicing, or channel phosphorylation. Auxiliary subunits of voltage gated ion channels can increase sodium channel functional diversity and affect channel biophysical properties as well as trafficking and surfacing (Tseng et al., 2007). Furthermore, channels expression and function are also regulated by phosphorylation (Scheuer, 2011). Therefore, in addition to postnatal increases of channel expression levels multiple additional mechanisms could in principle increase GF axonal conduction velocity during the first day post-eclosion. A direct proof for increased sodium channel expression levels as the cause for GF speeding would require single-cell qRT-PCR of FISH.

### L-type calcium channels are required for postnatal increases in GF axonal conduction velocity

Our data indicate that sodium channels account only partially for GF axonal conduction velocity increases. Targeted RNAi knock-down of *DmCa1D* L-type calcium channels in the GF strongly decreased axonal conduction velocity at both stages tested, and it abolished postnatal speeding almost completely. An acute function of Dmca1D in fast AP conduction is difficult to reconcile with the slow activation kinetics of most L-type channels (Mermelstein et al., 2000; Yasuda et al., 2003). But note that L-type channel activation kinetics can be altered by alternative splicing and auxiliary subunits (Birnbaumer et al., 1998; Lipscombe et al., 2002; Liu et al., 2004). Ten different Dmca1D isoforms are annotated, and *Drosophila* HVA channels interact with auxiliary β- and α2δ-subunits. In larval MNs calcium influx through Dmca1D indirectly reduces AP duration, rise and decay times, and refractory period (Kadas et al., 2017), parameters which vary inversely with conduction velocity in mammalian unmyelinated fibers (Paintal, 1967; Swadlow and Waxman, 1976). Therefore, axonal L-type channels may acutely increase GF conduction velocity.

Alternatively, activity dependent calcium influx through L-type channels may regulate the expression levels of other ion channels (Flavell and Greenberg, 2008), but this possibility is difficult to reconcile with the much stronger effect of Dmca1D RNAi at 24 h as compared to 1 h post-eclosion. However, a reduction of Dmca1D expression by targeted RNAi expression critically counteracts the normal increases in GF conduction velocity. Postnatal regulation of L-type channel expression has also been reported in spinal cord (Jiang et al., 1999) and sinoatrial node (Protas et al., 2001). At this point we cannot pinpoint whether Dmca1D current has a direct effect on conduction velocity, or whether calcium influx through Dmca1D channels alters the transcriptional level or properties of other ion channels, thus affecting GF conduction velocity indirectly. Ideally, one would measure expression levels of multiple channels with and without Dmca1D RNAi at both stages, but single-cell qRT-PCR or FISH are beyond the scope of this study.

### Shaker and BK channels increase GF conduction velocity and postnatal speeding

Outward potassium currents play crucial roles in limiting sodium channel inactivation or promoting de-inactivation (Baranauskas, 2007). In mammals and *Drosophila*, the fast AP afterhyperpolarization depends on A-type and BK potassium channels (Lancaster and Nicoll, 1987; Sah and Faber, 2002; Kadas et al., 2015). In the GF targeted RNAi knock-down of different potassium channels had different effects (Table 2): Shal-RNAi (*Kv4* homolog) had only small effects on conduction velocity and speeding. Slo-RNAi (BK homolog) reduced conduction velocity at both stages and reduced speeding by ∼25%. Shaker-RNAi (*Kv1* homolog) reduced axonal conduction speed only in mature flies, thus limiting postnatal speeding. Shaker channels localize to the axons of *Drosophila* central neurons (Rogero et al., 1997), motoneurons (Ryglewski and Duch, 2009), and likely also the GF axonal membrane (this study). In Shaker mutants APs recorded from the GF axon showed a prolonged repolarization (Tanouye et al., 1981; Tanouye and Ferrus, 1985). Slowpoke channels sharpen AP shape and shorten refractory period in *Drosophila* motoneurons (Kadas et al., 2015, 2017). Hence, Shaker and Slo are candidates for increasing GF axonal conduction velocity.

Given that *shaker* RNAi knock-down affects GF axonal conduction velocity at 24hPE but not at 1hPE, it seems likely that Shaker channel expression and localization to the GF axonal membrane is upregulated during the first day of adult life. Postnatal up-regulation of Shaker has also been described in rat neocortical and TG neurons, sympathetic neurons and in mouse hippocampus (McFarlane and Cooper, 1992; Bordey and Sontheimer, 1997; Seifert et al., 1999; Grosse et al., 2000; Guan et al., 2011). However, on the level of immunocytochemistry for Shaker channels that were endogenously tagged with GFP, we could not find any evidence for a significant upregulation of Shaker protein levels, neither in the GF axonal membrane nor in neighboring descending axons. One possible explanation is that newly emerged flies (1hPE) lack auxiliary subunits (e.g., hyperkinetic) that render Shaker channel functional, or that other co-factors increase Shaker current through a given amount of channels during the first day of adult life. Although the precise mechanism requires further study, our data show that postnatal regulation of Shaker is required for postnatal increases of AP conduction velocity in the GF.

In sum, fast sodium current through Para channels, L-type calcium current through Dmca1D channels as well as A-type potassium current through Shaker and BK current through Slowpoke channels all increase axonal AP conduction velocity, most likely through AP sharpening. Postnatal regulation of either the expression levels (including appropriate trafficking to plasma membrane) or the function of these channels during the first day of adult life co-operatively increases AP conduction velocity by 80%, but the relative contributions of each channel are different. Regulating the expression levels and/or properties of axonal ion channels provides a valuable means for increasing the speed of information transfer in unmyelinated axons, and thus, is likely a critical factor in invertebrate NS development and evolution. Therefore, direct measures of transcript levels or studies of mechanisms (e.g., differential splicing, post-translational modification, auxiliary subunits) controlling ion channel function, during postnatal maturation, will be needed to discriminate among these possibilities. By contrast, in the vertebrate NS myelination provides a more effective means, and thus mature conduction velocity of the unmyelinated GF remains ∼20–60 times slower than that of vertebrate Aβ sensory axons and α motoneurons, despite roughly similar diameters.

## Synthesis

Reviewing Editor: Miriam Goodman, Stanford University Decisions are customarily a result of the Reviewing Editor and the peer reviewers coming together and discussing their recommendations until a consensus is reached. When revisions are invited, a fact-based synthesis statement explaining their decision and outlining what is needed to prepare a revision will be listed below. The following reviewer(s) agreed to reveal their identity: NONE. Note: If this manuscript was transferred from JNeurosci and a decision was made to accept the manuscript without peer review, a brief statement to this effect will instead be what is listed below.

Thank you for forwarding this revised manuscript. You have addressed the reviewer's main criticisms by revising the text rather than by providing additional data regarding: RNAi efficiency, ion channel gene expression, axon diameter. In your rebuttal, you carefully outline the possibilities regarding the change in ion channel activity. Please revise the discussion to more clearly indicate the possible mechanisms (e.g. transcription, trafficking, post-translational modification) and to communicate to readers that additional data, such as direct measures of transcript levels would be needed to discriminate among these possibilities.
